# Supplementary material for: Genome-wide identification of ZmHMAs and association of natural variation in ZmHMA2 and ZmHMA3 with leaf cadmium accumulation in maize
Source: PeerJ. 2019 Oct 23;7:e7877. doi: 10.7717/peerj.7877 (PMC6815194; doi:10.7717/peerj.7877)
Supplement: Figure S2 [file peerj-07-7877-s010.pdf]

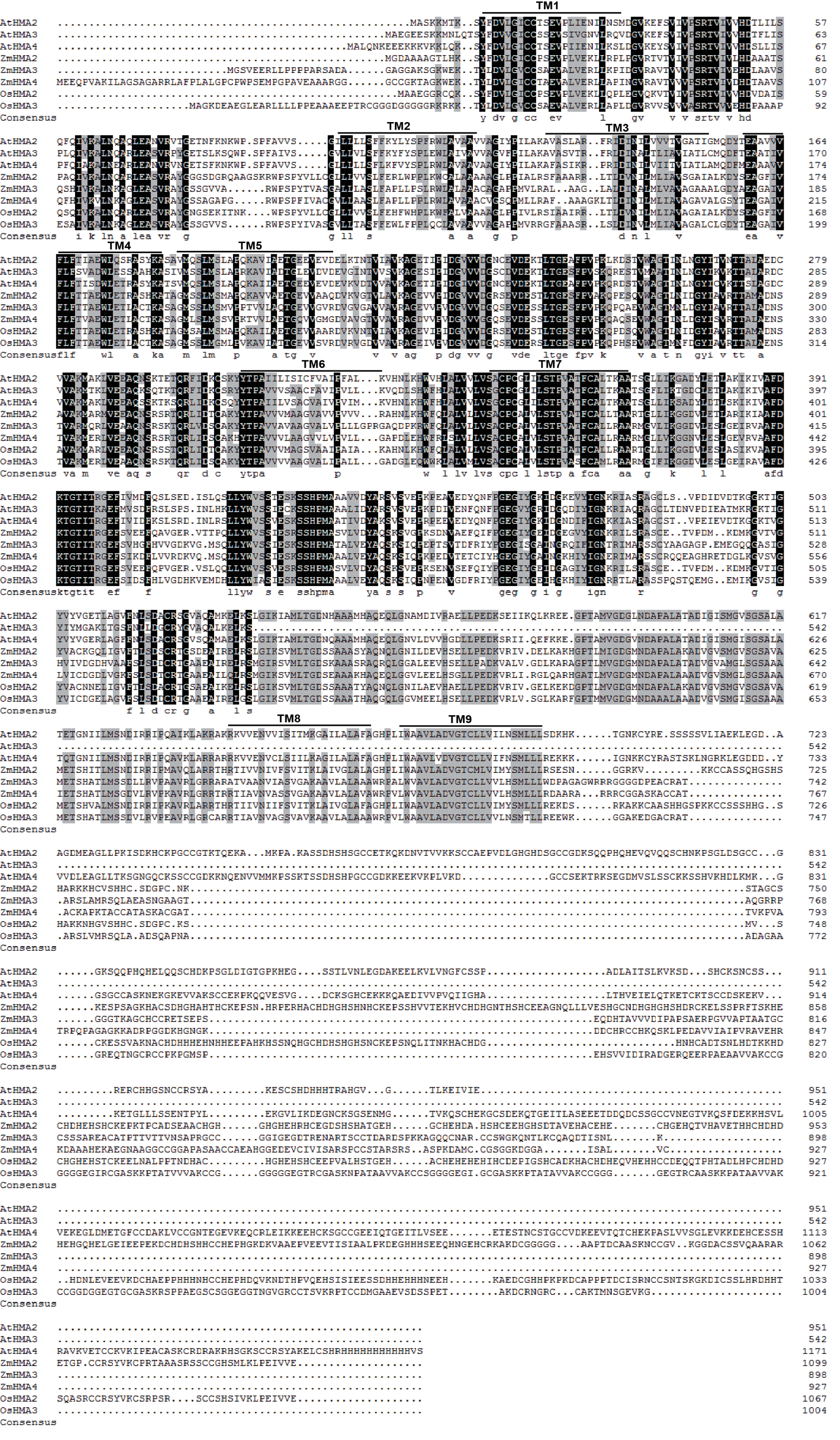
TM1
TM2
TM3
TM4
TM5
TM6
TM7
TM8
TM9
AtHMA2
AtHMA3
AtHMA4
ZmHMA2
ZmHMA3
ZmHMA4
OsHMA2
OsHMA3
Consensus
