# Supplementary material for: Genome-wide identification of ZmHMAs and association of natural variation in ZmHMA2 and ZmHMA3 with leaf cadmium accumulation in maize
Source: PeerJ. 2019 Oct 23;7:e7877. doi: 10.7717/peerj.7877 (PMC6815194; doi:10.7717/peerj.7877)
Supplement: Figure S1 [file peerj-07-7877-s009.pdf]

**MOTIF-1**	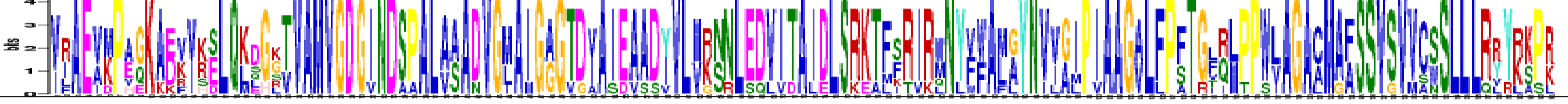
**hydrolase domain**	[VI][RI]AE[VA][KM]P[AE][GQ]KA[DE][VK][VI][KR][ES]LQ[KL]DG[GK][TV]VAMVGDGINDSPAL[AV][AS]ADVG[ML]AIG[AG]GTD[IV]AIEAAD[IY]VL[MV][KR][NS]NLEDVITAIDLSRKTF[SF]RIR[WM]NY[FV][WF]A[LM][AG]YNV[VI][GA]IPIAAG[AV]LFP[FS]TG[FL][QR][LM]PPW[LV]AGA[CA]MA[FA]SSVSVVC[SW]SLLLR[RY]Y[KR][KS]P[RK]
**MOTIF-2**	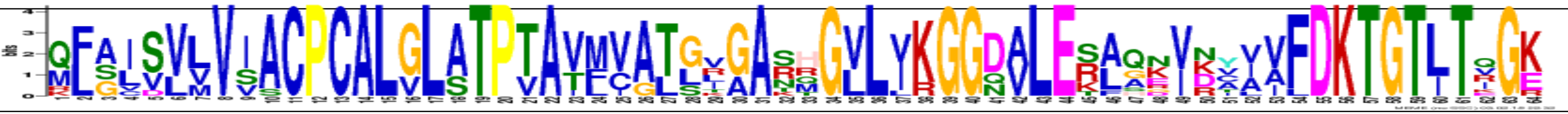
**E1-E2_ATPase**	[QM][FL][AGS][IL][SV][VL][LV]V[IS]ACPCAL[GV]L[AS]TP[TV]A[VT][MF][VC]A[TL][GL]V[GA]A[SR]HG[VL]L[IV]KGGD[AV]LE[RS][AL]Q[KN][VI][DKN]Y[VAI][VA]FDKTGT[LI]TQG[KE]
**MOTIF-3**	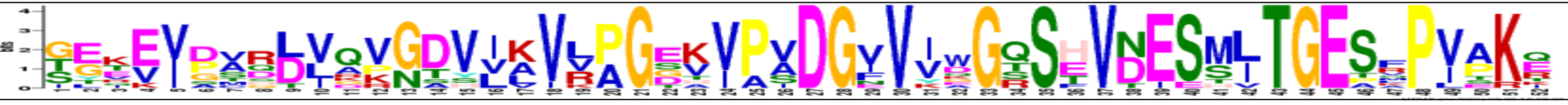
**E1-E2_ATPase**	[GST][EG]K[EV][VI][DP][AV]R[DL][VL]QV[GN]DV[ILV][KA]V[LRV][PA]GE[KV][VI]P[VA]DGVV[IV]WGQS[HE]V[DN]ES[MS][LI]TGESFP[VI][AP]K[EQ]
**MOTIF-4**	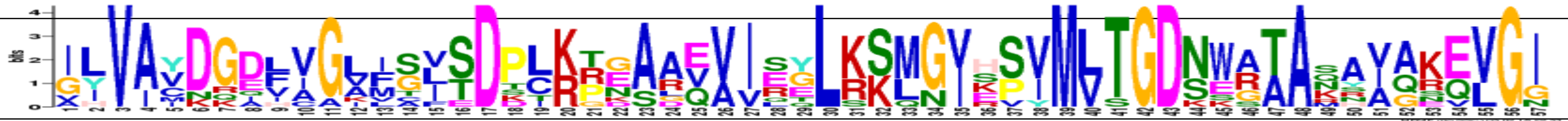
**Hydrolase domain**	[IG][LIY]V[AI][VYC]DG[DE][FLV][VAI]G[LAV][FIM][SG][ILV][ST]DP[LC][KR][PRT][EGN][AS][AR][EQV][VA][IV][ESR][GYE]L[KR][SK][ML][GN][IV][HEKS][SP][VI]M[LV]TGD[NS][WE][ARG][TA]A[KNQS]A[VAI][AQG][KR][EQ][VL]G[IG]
**MOTIF-5**	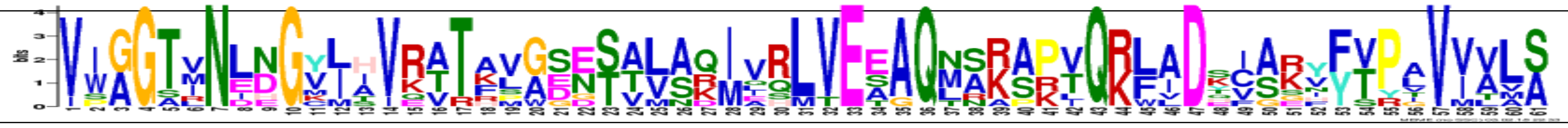
**E1-E2_ATPase**	V[IW][GA]GT[VIM]N[LE][ND]G[VY][IL][HAI]V[RK][ATV]T[AFK][VL][GA][SDE][EN][ST][ATV][LV][AS][QKR][IM]V[RQ]LVE[EAS]AQ[MNL][SA][KR][AS][PKR][VT]Q[RK][LF][AI]D[KS][CIV][AS][KR][YV][FY][TV]P[AL]V[VI][VA]L[SA]
**MOTIF-6**	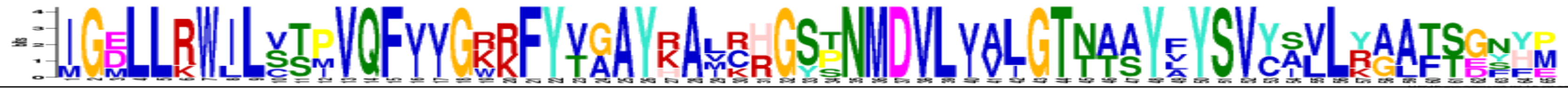
**Unknown**	[IM]G[DEM]LL[RK]W[IL]L[SVC][TS][PIM]VQF[VI][VI]G[KRW][RK]FY[VT][GA]AY[KRH]A[LIMV][CRK][HR]G[SY][PTS]NMDVL[VI][AV][LI]GT[NT][AT][AS]Y[FALV]YSV[YC][ASI][VL]L[RYK][AG][AL][TF][ST][GDE][NFSY][HYF][MPE]
**MOTIF-7**	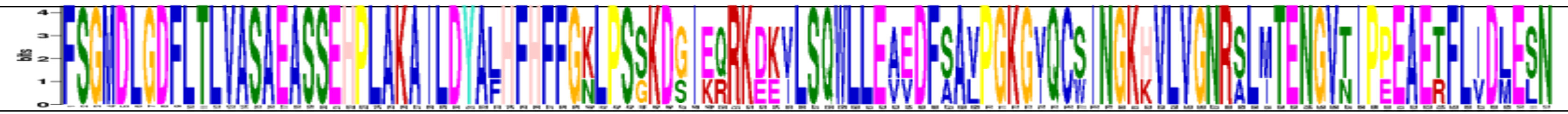
**Unknown**	FSGMDLGDFLTLVASAEASSEHPLAKAILDYA[LF]HFHFFG[KN]LPS[SG]KD[GS]I[EK][QR]RK[DE][KE][VI]LSQWLLE[AV][EV]DF[SA]A[VL]PGKG[VI]QC[SW]INGK[HK]VLVGNR[SA]L[MI]TENGV[TN]IP[PE]EAE[TR]FL[IV]D[LM]E[SL]N
**MOTIF-8**	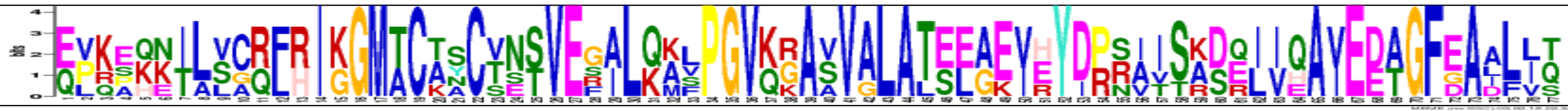
**Heavy-metal-associated domain**	[EQ][PVL][KQR][EAPS][KQH][KNE][IT][LA][SVL][CAG][RQ][FL][RH]I[KG]GM[TA]C[ATK][SANY]C[TVS][NES][ST]VE[GFRS][AI]L[QK][AKM][LFSV]PGV[KQ][GRK]A[SVA]V[AG]LA[TL][ES][EL][AG][EK][VI][EHR]Y[DI][PR][RSN][AIV][ITV][ST][AKR][DS][EQR][IL][IV][QEH]A[VI]E[DE][AT]GF[EDG]A[ADIL][LF][ILV][QTS]
**MOTIF-9**	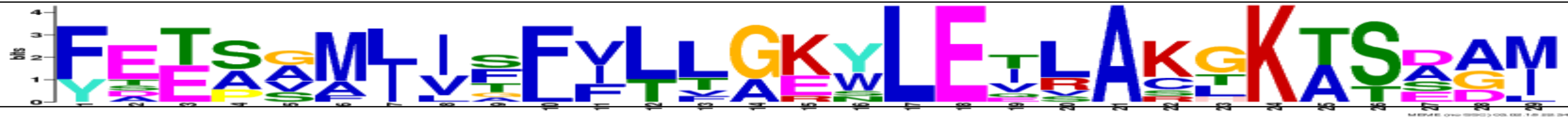
**Unknown**	[FY]E[ET][SAP][AGSV][MA][IL][IV][FST][FL][VFI][LT][LT][GA][KE][YW]LE[ITV][LR]A[KC][GLT]K[AT][ST][ADES][AGD][MI]
**MOTIF-10**	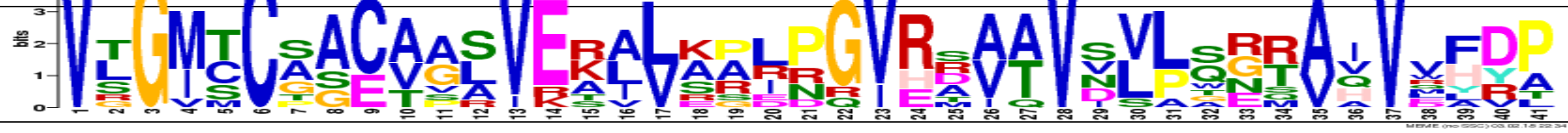
**Heavy-metal-associated domain**	V[LTS]G[MI][TCS]C[ASG][AGS][CE][AVT][AG][SLA]VE[KRA][ALI][LV][AKS][APR][LIR][PNR]G[VI][REH][ADRS][AV][AT]V[SDNV][VL][LP][QS][GREN][RT][AV][IHQV]VV[FH][DRY][PA]
